# Ultrasound-guided minimally invasive thread release of carpal tunnel: a cadaveric study

**DOI:** 10.1007/s11547-025-01952-w

**Published:** 2025-01-21

**Authors:** Suren Jengojan, Philipp Sorgo, Alessio Piacentini, Johannes Streicher, Domenico Albano, Gregor Kasprian, Veith Moser, Gerd Bodner

**Affiliations:** 1 Department of Biomedical Imaging and Image-Guided Therapy, Division of Neuroradiology and Musculoskeletal Radiology, Medical University of Vienna, Waehringer Guertel 18-20, 1090 Vienna, Austria; 2 IRCCS Istituto Ortopedico Galeazzi, Universita’ Degli Studi Di Milano, Milan, Italy; 3 Department of Anatomy and Developmental Biology, Karl Landsteiner University of Health Sciences, Dr.-Karl-Dorrek-Straße 30, 3500 Krems an Der Donau, Austria; 4 Department of Trauma Surgery, Lorenz Boehler Hospital, Vienna, Austria; 5 Neuromuscular Imaging Ordinationszentrum Döbling, Heiligenstädter Straße 46-48, 1190 Vienna, Austria

**Keywords:** Ultrasonography, Carpal tunnel syndrome, Decompression, Wrist, Cadavers

## Abstract

**Purpose:**

Thread release of the carpal tunnel is the most recent of several minimally invasive ultrasound-guided carpal tunnel release techniques. The purpose of this article is to provide a step-by-step guide for minimally invasive, ultrasound-guided thread release of the carpal tunnel focused on transecting the transverse carpal ligament with minimal damage to the palmar aponeurosis on anatomical specimens.

**Methods:**

Fifteen ultrasound-guided carpal tunnel thread releases were performed on the wrists of soft-embalmed anatomical specimens, which were dissected immediately after the intervention. The procedures were performed by two musculoskeletal radiologists with 25 and 8 years of experience, respectively, in interventional radiology. Ultrasound visibility, completeness of transection, and damage to surrounding structures were evaluated on a score from 1 to 3.

**Results:**

We achieved a complete transection of the transverse carpal ligament in 11 of 15 interventions (73%) and an incomplete transection in the remaining four (27%). No neural or vascular structures were harmed. In two cases (13%), there was irrelevant damage to flexor tendons. The ultrasound visibility was rough in five specimens (33.3%), moderate in five (33.3%), and optimal in five (33.3%). Essential structures were delineated in all cases.

**Conclusion:**

Thread release of the carpal tunnel leads to only minimal damage to skin, as well as structures within the carpal tunnel and the palmar aponeurosis, promising a low amount of postinterventional complications.

**Relevance statement:**

Our study showed that minimally invasive ultrasound-guided thread release of the carpal tunnel is a feasible approach in the anatomical model. The results may provide a basis for further research and refinement of this technique.

**Graphical abstract:**

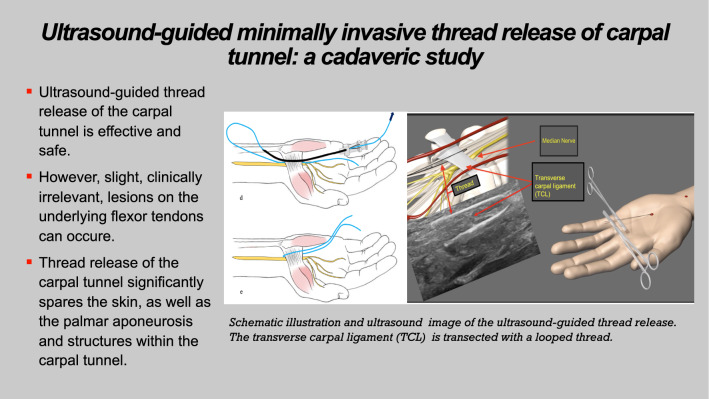

**Supplementary Information:**

The online version contains supplementary material available at 10.1007/s11547-025-01952-w.

## Key points

- In CTS patients, the transverse carpal ligament often needs to be transected.
- Thread release of the carpal tunnel is effective and safe.
- Thread release of the carpal tunnel considerably spare the skin, as well as structures within the carpal tunnel and the palmar aponeurosis.
- Ultrasound-guided thread release is a potential treatment alternative in CTS patients.

**Supplementary Information:**

The online version contains supplementary material available at 10.1007/s11547-025-01952-w.

## Introduction

With a prevalence of approximately 3.8% in the general population, carpal tunnel syndrome (CTS) is the most common entrapment neuropathy, posing a global health burden [1, 2]. Compression of the median nerve at the level of the wrist (carpal tunnel) results in dysesthesias, pain, and, in more severe cases, atrophy of the median nerve-innervated muscles. Essential for the diagnosis of CTS area precise medical history and clinical examination nerve conduction studies and high-resolution ultrasound (HRUS) [4, 5]. HRUS depicts morphological changes in the median nerve [6, 7]. In addition, surrounding soft tissues (flexor tendons, transverse carpal ligament, nerves, and vessels) can also be assessed in detail [8]. Mild to moderate CTS can be treated conservatively, whereas more severe cases require surgical or minimally invasive ultrasound-guided percutaneous decompression [3, 9]. Although open and endoscopic surgical approaches for carpal tunnel release (CTR) are available [3, 10, 11], complications include pillar pain, scar tenderness, recurrent CTS [12], trigger finger [13], and Dupuytren’s contracture (DC). The correlation between surgical approaches and DC was first noted by Wroblewski in 1973 in a small report of seven patients, with no further studies exploring it, but it confirms our frequent observations [14]. Although the exact etiopathogenesis remains unclear, research suggests that DC is linked to immune-mediated microvascular injury, leading to abnormal fibroblast proliferation and extracellular matrix protein production due to altered pro-inflammatory and pro-fibrotic cytokines. Studies also show reduced collagen deposition in patients treated with anti-TGF-β antibodies [15–17]. Repeated microtraumas, including surgical trauma, may exacerbate this process. Therefore, minimizing tissue injury and the subsequent inflammatory response during surgery would be ideal.

Recently, ventures to develop ultrasound-guided minimally invasive techniques were conducted with the goal of reducing surgical trauma and postoperative complications. Several different types of cutting instruments have been utilized, in the form of hook knives [18], needles [19], and sawblades [20, 21]. A promising approach was proposed by Guo et al. in 2015 [22]. This minimally invasive thread release technique has already been tested for releasing various entrapment neuropathy sites, including the Guyon canal, the cubital tunnel, and for the release of A1 pulleys in cases of stenosing tenosynovitis [23–25].

Recent clinical studies have shown a fast recovery period after ultrasound-guided thread CTR (TCTR). Mende et al. reported an average of 5.2 days before return to daily activities, five times faster than after open surgery and 1.5 times faster than using a hook knife [26]. Guo et al. described a significant relief of symptoms after 3–5 hours and the ability to use the hands for simple daily activities on the same day [27]. A faster return to work without restrictions (12 as opposed to 33 days) after TCTR compared to open techniques has also been reported by Asserson et al. [28]. An initial study concerning the long-term outcome of TCTR 2 years after the intervention has described no adverse effects in a group of patients that included those with severe CTS and anatomical variants [29]. The purpose of our study was to assess the efficacy and safety of TCTR in an anatomical specimen model. In addition, emphasis was placed on the anatomy and potential injury of the palmar aponeurosis (PA), as we suspect that injury to the PA could lead to longer convalescence after CTR. We hypothesized that TCTR would be an effective and safe method for transecting the TCL, while minimizing trauma to the surrounding tissues.

## Materials and methods

The study was submitted to and accepted by the local committee for scientific integrity and ethics of the Karl Landsteiner Private University (vote number: 1052/2021). All procedures were performed in compliance with relevant laws and institutional guidelines. Informed consent regarding the use of their physical remains was obtained from all body donors.

A single-center, prospective, anatomical study was conducted on the hands of 15 Thiel-embalmed [13] or fresh frozen [2] cadavers.

All study-related procedures were conducted at the Department of Anatomy and Developmental Biology of Karl Landsteiner University for Health Sciences in St. Pölten, Austria, between June and November 2021. No complex statistical analysis was required for this study.

Selection was based on availability. Exclusion criteria were signs of injury or surgery in the region of the wrist or hand. No specimens had to be excluded.

We performed fifteen TCTR (13 left and two right hands). The interventions were performed by two musculoskeletal radiologists, G.B. and S.J. (25 and 8 years of experience in musculoskeletal interventional ultrasound, respectively). Subsequently, the hands and wrists were dissected to assess the transection of the TCL and potential injury to adjacent structures. Dissection and assessment of the anatomical situs was done by J.S. and P.S. (36 and 4 years of experience in anatomical dissections, respectively). The interventions were performed using a 20-gauge 10 cm spinal needle and a 22-gauge braided stainless steel thread (Core Essence Orthopaedics). To enhance the visibility of the thread’s path during dissection, the threads were embedded in a bath of histological hematoxylin–eosin stain for several hours prior to the intervention. Continuous ultrasound guidance and visualization of anatomical structures was provided by a General Electronics Logiq E10s musculoskeletal ultrasound system with high-frequency (6-22 MHz) broadband linear probes (GE Healthcare, Milwaukee, USA).

### Technical execution

**Fig. 1:**
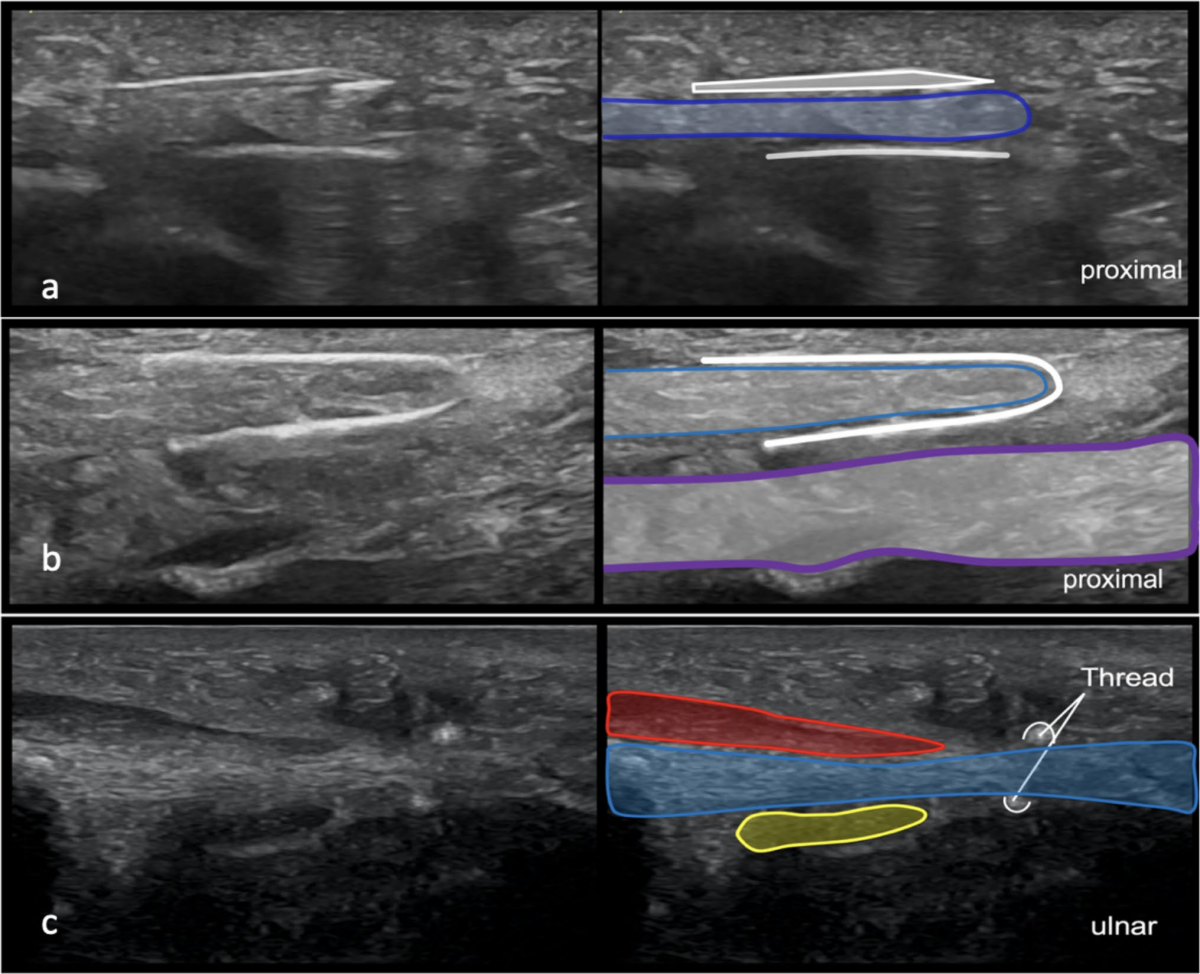
**a** Placement of the needle above the TCL |white: needle and cutting thread and blue: TCL. **b** Final position of the thread in the long axis │ blue: TCL; white: cutting thread; green: palmar aponeurosis; and purple: flexor tendons inside the carpal tunnel. **c** Final position of the cutting thread in the short axis. │ red: thenar musculature; blue: TCL; and yellow: median nerve

**Fig. 2:**
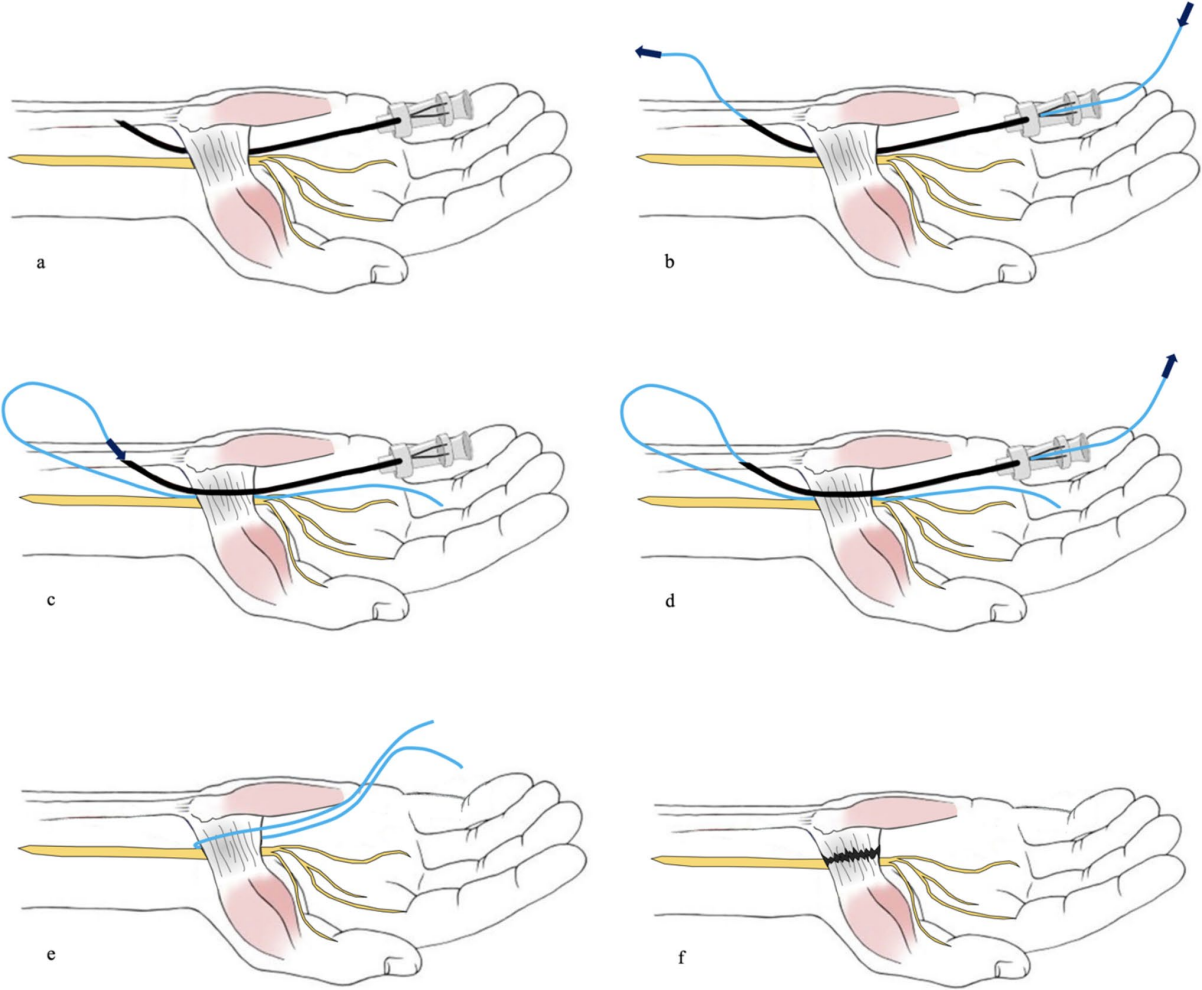
Minimally invasive ultrasound-guided thread release protocol; in the first step (**a**), a spinal needle is placed deep to the TCL, and the thread is inserted (**b**). Then, the needle is removed and reinserted superficial to the TCL, using the same incision hole (**c**). The thread is looped around the TCL (**d**), and the TCL is transected after removal of the needle (**e**, **f**)

**Fig. 3:**
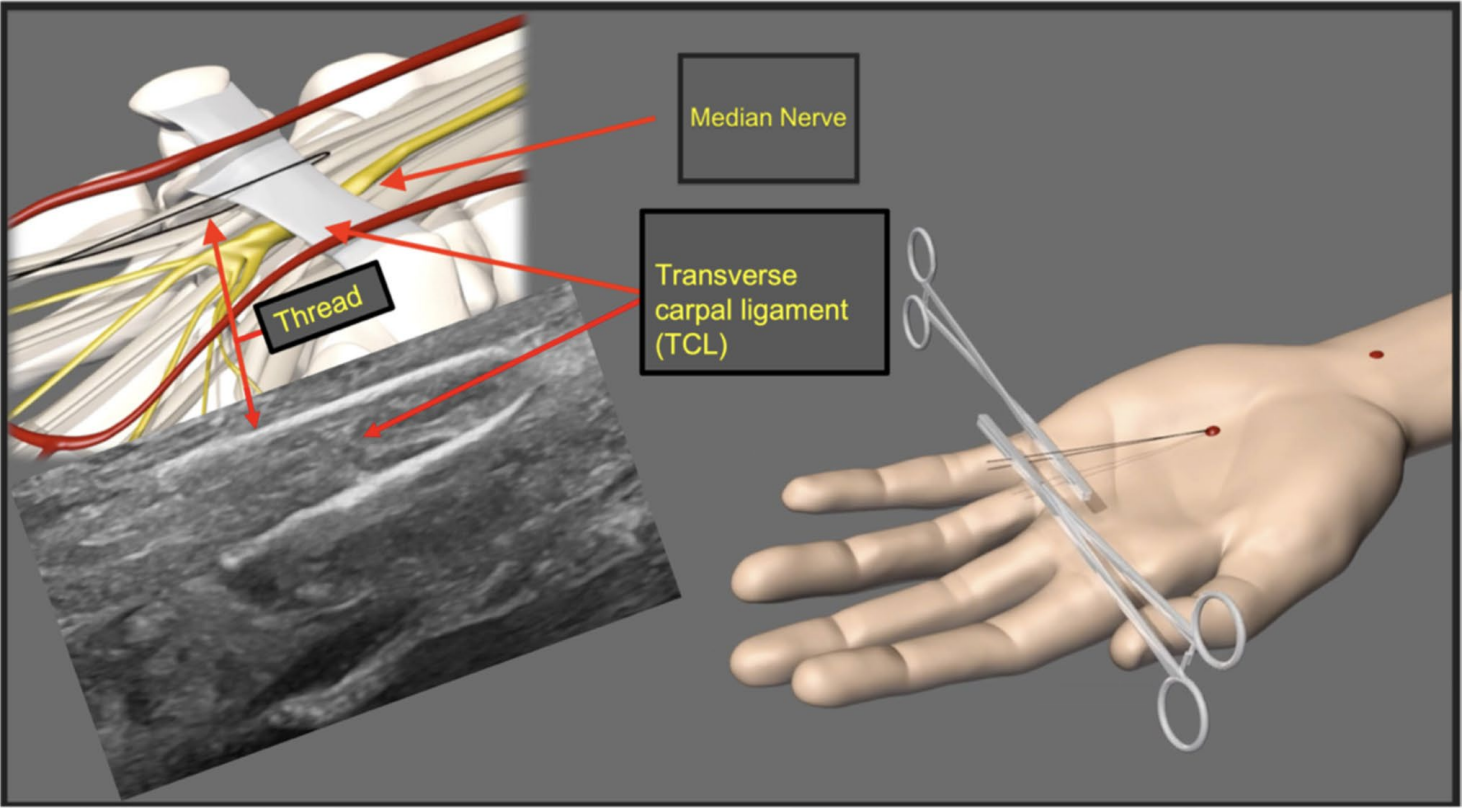
Final position of the cutting thread (long axis)

We utilized the modified protocol for TCTR described by Guo et al. [30]. Here, in detail, the intervention is demonstrated step-by-step (Fig. 1):

1. Under ultrasound guidance, a spinal needle (20-gauge, 100-mm-long spinal needle) was inserted distal to the TCL, approximately 0.5–1 cm proximal to the superficial palmar arch. Under continuous hydrodissection (injection of NaCl through the needle), the needle was advanced beneath the TCL toward the exit point proximal to it.
2. The cutting thread (a commercial, medical-grade, stainless steel woven thread, with a high friction coefficient, 0.3 mm in diameter and 30 cm in length) was inserted distally into the needle and passed through to the proximal end.
3. The needle was removed, reinserted at the same insertion site, and advanced superficial to the TCL toward the same exit site. Hydrodissection was performed to separate subcutaneous tissue and the palmar aponeurosis from the carpal ligament (Fig. 2a).
4. The proximal end of the thread was inserted into the needle tip, and a loop around the TCL was formed by passing the thread through the needle.
5. The needle was then removed.
6. After confirming the correct loop placement via HRUS control, the TCL was transected by applying alternating pulling forces on the ends of the thread, using the thread as a sawblade (Fig. 2b, 2c).
7. Following the procedure, only two small needle incision holes were visible on the skin.
8. The TCL was then transected by applying alternating pulling forces, using the thread similarly to a wire saw (Fig. 3).

The video attached in the Supplementary Material (Video 1) shows the procedure in an animation, as well as the corresponding ultrasound images.

### Assessment and documentation

We assessed each intervention with regard to three parameters: ultrasound visibility; success of transection; and injury to adjacent structures (nerves, tendons, and vessels). Each of these parameters was graded on a three-point scale [24]. Assessment of outcome and injury to adjacent structures was performed and discussed between all authors after the dissection, whereas ultrasound visibility was defined by both expert radiologists (G.B. and S.J.).

The exact duration of the procedure was not measured exactly but was approximately 10 min. This did not include pre- and postinterventional procedures that would be performed in a clinical setting.

No differences were observed in the results between the two radiologists with different levels of experience.

Ultrasound images and videos were saved using the implemented saving system of the ultrasound device. Furthermore, the dissected specimens were photographically documented throughout the procedure**.**

## Results

**Fig. 4:**
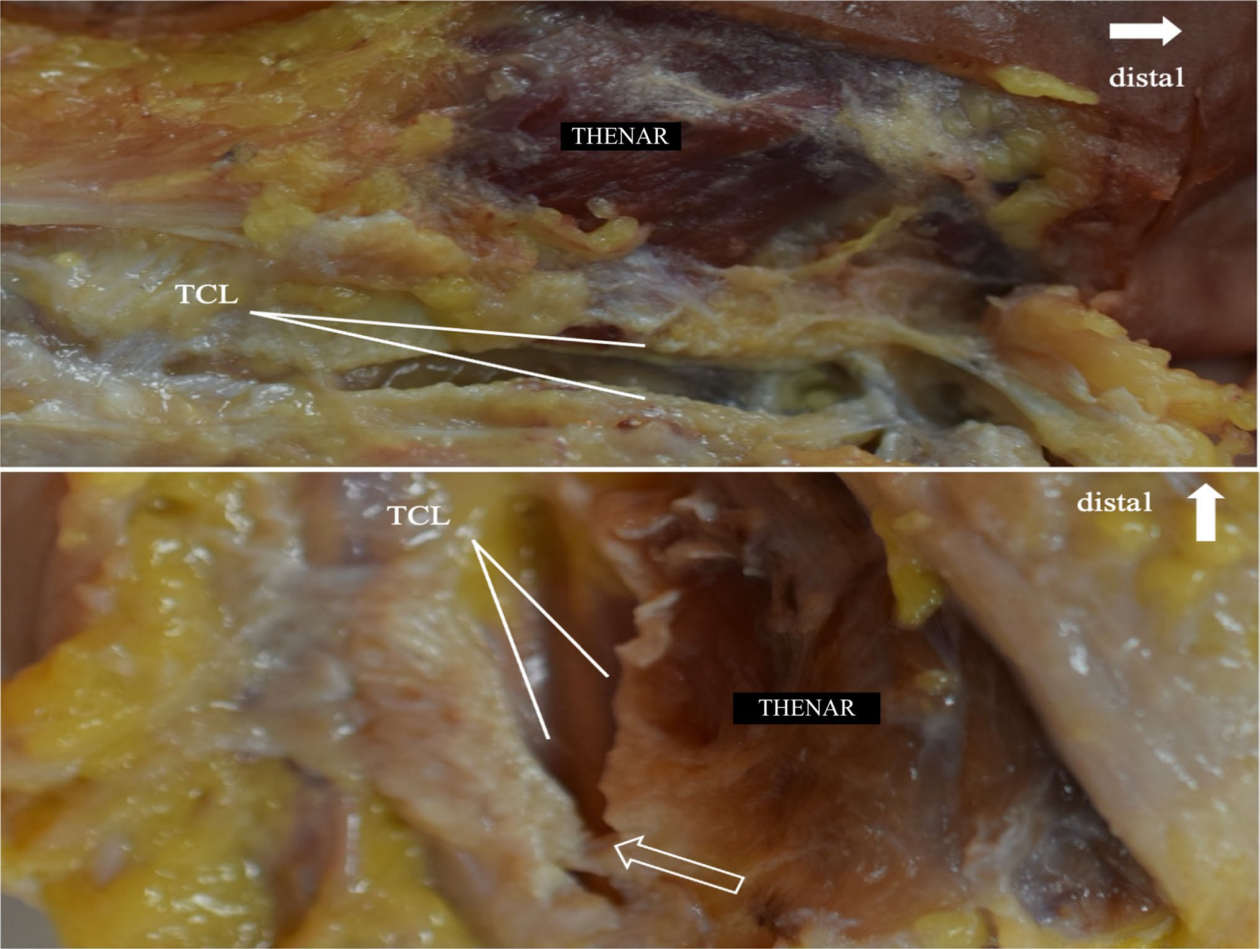
(above) Completely transected TCL on a left hand (3 on our score). │ MN median nerve and TCL transverse carpal ligament. (below**)** Partial transection of the TCL in a right hand (2 on our score) │ void arrow: small remainder of the TCL; TCL transverse carpal ligament

**Fig. 5:**
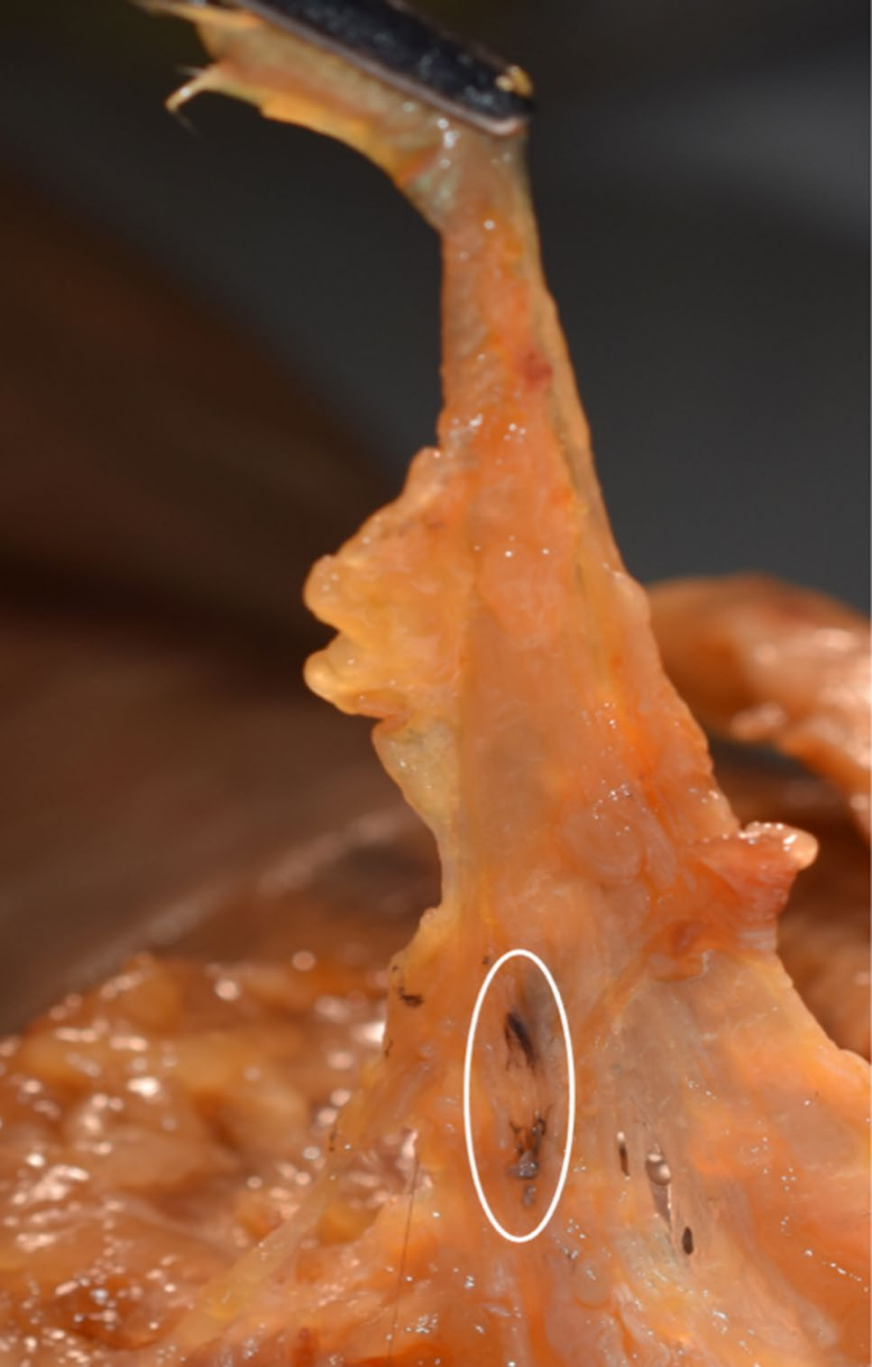
Palmar aponeurosis (pulled upward) showing only minor injury at the point of needle insertion (oval), recognizable by the colored trace of the cutting thread

The technical success rate was 100%, as the loop of the thread was successfully positioned around the TCL in all specimens. The technical efficacy was 73%, as the complete transection of the TCL (Fig. 4) was achieved in 11 of 15 specimens. Incomplete transection above 75% of the TCL (Fig. 5) was achieved in 20% (three of 15). In the one remaining case, only an incomplete transection below 75% of the TCL could be achieved. We used a 75% confidence margin because, in clinical practice, positive outcomes are often achieved even when the resection of the TCL is not complete. No macroscopically identifiable neural or vascular structures were injured in any specimen. Irrelevant, longitudinal, and superficial lesions to the flexor tendons were observed in two cases. Results are summarized in Table 1 (supplementary material).

If a long palmar muscle (LPM) existed (five cases), only minimal injury was caused to the palmar aponeurosis (AP) by the insertion site of the needle (Fig. 5). This occurred when navigating the needle medially to the tendon of the LPM and AP.

In the absence of the LPM, the AP was fused with the TCL, resulting in slightly greater injury to the AP. However, it could be kept to a minimum due to ultrasound-guided navigation and hydrodissection.

Ultrasound visibility varied between specimens. Poor visibility was observed in five cases (33.3%). Of these, there was an incomplete transection above 75% in two cases (Case 4 and Case 6).

Of the five cases (33.3%) with average ultrasound visibility, there was an incomplete transection above 75% in Case 7 and irrelevant injury (i.e., very superficial and short distance tendon or tendon sheath scratches) to a flexor tendon in Case 8. Of the remaining five cases (33.3) with good ultrasound visibility, Case 13 showed an incomplete transection below 75%, and Case 5 showed irrelevant Injury to a flexor tendon. There was good ultrasound visibility and a complete transection in both fresh frozen specimens, one of which (Case 5) showed irrelevant flexor tendon injury.

## Discussion

We performed TCTR on 15 cadaveric specimens. Our results suggest that TCTR is an effective and safe technique. We achieved a rate of successful transection of the TCL of 75%. Guo et al. reported a full transection of the TCL in 100% (11 of 11) of cases and [27] Burnham et al. in 64% (nine of 14) [31]. Both studies did not report any injury to neural or vascular structures. Due to constant real-time ultrasound guidance and hydrodissection, the impact on adjacent anatomical structures could be kept to a minimum. Injury to structures within the carpal tunnel was negligible, since, in only two cases, flexor tendons were superficially affected. Injury to the PA was either negligible (small puncture holes, see Fig. 9) or unavoidable regardless of the type of CTR because of the fusion of the palmar aponeurosis with the TCL due to the absence of the long palmar muscle. While ultrasound visibility varied between different specimens, all interventions were technically possible. The percentage of complete transections was lowest in the group with poor ultrasound visibility (60%), compared to average and good visibility (80% each). Although there is no clear reason for the one incomplete transection in the good visibility group, it is possible that thin anatomical structures, such as the TCL in some embalmed arms, exhibit incomplete or reduced delineation in ultrasound, making precise transection more difficult. One case each (20%) in the high and medium visibility group showed irrelevant flexor tendon injury. It would seem plausible that better ultrasound visibility might lead to better results; however, studies with a higher sample size would be necessary to verify this. Injury to surrounding tissue and skin was minimal, but low ultrasound visibility might lead to insufficient delineation between anatomical structures, especially in cases with anatomical variants or abnormalities. It is essential to sufficiently visualize any structures at risk before and during the procedure, foremost the median nerve and its branches. Therefore, it is the authors’ opinion that TCTR should be performed only by physicians familiar with interventional sonography and also with possible anatomical variants. Ultrasound visibility may vary in different patients, for example, in adipose or exsiccated individuals. This could, under certain circumstances, be a contraindication for the intervention.

We have shown that not only is the injury to structures within the carpal tunnel negligible, but also that the injury to the AP is minimal. In fact, using a lesser diameter instrument (compared to an endoscope or hook knife) in the confined space of the CT allows minimal injury and trauma to the skin and to the subcutaneous soft tissue. Therefore, this may reduce the activation of the pro-inflammatory and pro-fibrotic cytokines around the AP (reflecting, at least partially, the hypothesis of the origin of DC) and resulting in a reduction of excessive scar formation, which, in some cases, has resulted in DC in patients after open surgery. This circumstance could also explain the fast postinterventional recovery described in recent clinical studies.

Our study faced several limitations. The limited availability of anatomical specimens restricted us from performing a higher number of interventions, leading to a small sample size insufficient for further statistical analysis. Due to privacy protection of the body donors, we did not know their exact age or previous health conditions. As this was a postmortem study, we cannot easily extrapolate our results to live patients. However, Thiel-embalmed cadavers have been shown in the past to be a suitable model for ultrasound-guided interventions [32–34]. It is our opinion that the average ultrasound visibility would still be superior in vivo and that the use of color Doppler sonography would be helpful for the detection of blood vessels.

During dissection, we observed a higher rate of the absence of the LPM (66.7%) than what has been described in the literature (1.5–63%) [35]. This is most likely due to the small sample size. However, given that the majority of the population has the palmaris longus tendon, this generally implies a less invasive approach for most individuals.

In conclusion, this study showed that TCTR is a suitable technique for the release of the carpal tunnel in an anatomical model. Unnecessary injury to adjacent structures could be avoided. However, further studies are recommended that include sufficient anatomical variants to further evaluate the safety of TCTR.

## Supplementary Information

Below is the link to the electronic supplementary material.

Supplementary file1 (DOCX 112 KB)

Supplementary file 2 (MP4 32,708 KB)

## Abbreviations

CT: Carpal tunnel
HRUS: High-resolution ultrasound
CTS: Carpal tunnel syndrome
CTR: Carpal tunnel release
TCTR: Thread carpal tunnel release
TCL: Transverse carpal ligament
AP: Palmar aponeurosis
LPL: Long palmar muscle
