# Supplementary material for: Ultrasound-guided minimally invasive thread release of carpal tunnel: a cadaveric study
Source: Radiol Med. 2025 Jan 21;130(4):524–33. doi: 10.1007/s11547-025-01952-w (PMC12008055; doi:10.1007/s11547-025-01952-w)
Supplement: Supplementary file 1 — Supplementary file1 (DOCX 112 KB) [file 11547_2025_1952_MOESM1_ESM.docx]

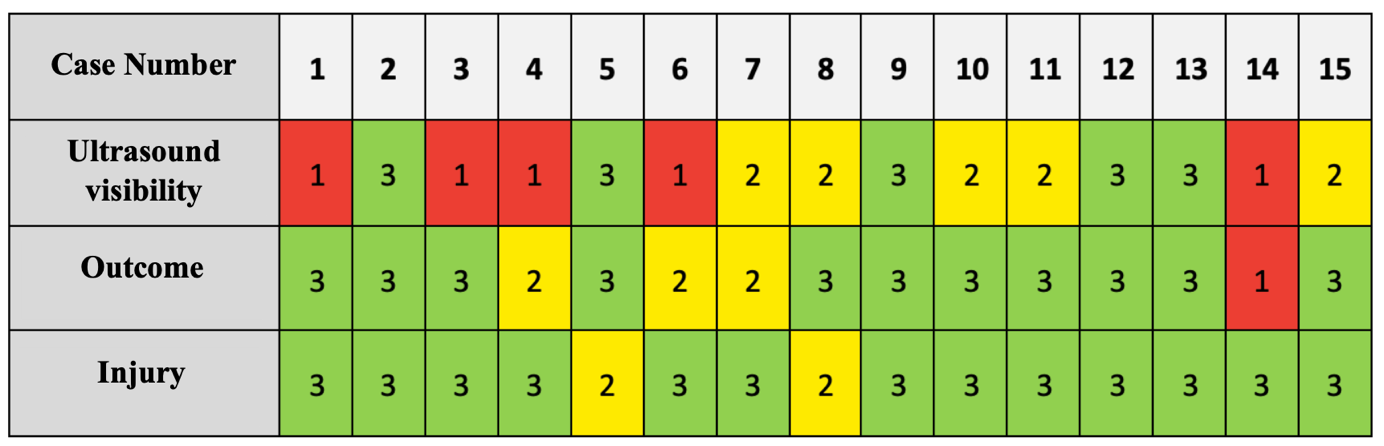


***Table 1*** *Cross-tables depict the results of 15 interventions using the scoring system graded on a three-point scale.*
